# Scalable methanol-free production of recombinant glucuronoyl esterase in *Pichia pastoris*

**DOI:** 10.1186/s13104-019-4638-9

**Published:** 2019-09-18

**Authors:** C. G. Conacher, M. P. García-Aparicio, G. Coetzee, W. H. van Zyl, J. F. Gӧrgens

**Affiliations:** 1 Departments of Process Engineering, Stellenbosch University, Private Bag X1, Matieland, 7602 South Africa; 2 Departments of Microbiology, Stellenbosch University, Private Bag X1, Matieland, 7602 South Africa

**Keywords:** Glucuronoyl esterase, Cellobiose dehydrogenase, Methanol-free constitutive expression system, *Pichia pastoris*, Lignocellulose biorefinery enzymes, Enzyme bioprocess technology

## Abstract

**Objective:**

Glucuronoyl esterase (GE) is an emerging enzyme that improves fractionation of lignin-carbohydrate complexes. However, the commercial availability of GE is limited, which hinders the research of GE-based bioprocesses for its industrial application in lignocellulose biorefineries. This study evaluated a workable, cost-effective, and commercially scalable production strategy to improve the ease of GE-based research. This strategy consisted of a constitutive and methanol-free enzyme production step coupled with a two-step filtration process. The aim was to determine if this strategy can yield copious amounts of GE, by secretion into the extracellular medium with an acceptable purity that could allow its direct application. This approach was further validated for cellobiose dehydrogenase, another emerging lignocellulose degrading enzyme which is scarcely available at high cost.

**Results:**

The secreted recombinant enzymes were functionally produced in excess of levels previously reported for constitutive production (1489–2780 mg L^−1^), and were secreted at moderate to high percentages of the total extracellular protein (51–94%). The constant glycerol feed, implemented during fed-batch fermentation, lead to a decline in growth rate and plateaued productivity. Tangential flow ultrafiltration was used to concentrate cell-free enzyme extracts 5–6-fold, reaching enzyme activity levels (1020–202 U L^−1^) that could allow their direct application.

## Introduction

Glucuronoyl esterase (GE) is a recently defined carbohydrate esterase that has been isolated from several microorganisms [1–6]. GE has potential for production of biofuels and biomaterials in lignocellulose biorefineries [7–12]. GE cleaves alkali-labile bonds from the lignin-carbohydrate complexes at acidic pH [12]. Recent reports have proven that the removal of glucuronic acid branches from the hemicellulose significantly improved release of fermentable sugars for biofuels production [7, 8, 13]. Additionally, GE can produce bioactive molecules that can be used in the cosmetic and pharmaceutical sectors [12].

Despite the industrial potential of GE, very limited recombinant production has been reported. Pure commercial preparations of GE do not exist. This is an impedance in the development of GE-based bioprocesses, since each study must first produce and purify the enzyme before it is possible to determine its lignocellulosic degrading capabilities. Some commercial enzyme cocktails exist with low GE side activity ([14], this study), but these cocktails do not allow for the determination of specific catalytic roles of each enzyme. To harness the catalytic ability of GE, understand its specific role, and determine optimum dosages, pure enzyme preparations are required. Notably, the recent increase in publications which evaluate application of GE, show an urgent need for GE production [7–10, 12].

Considering the above restriction in GE availability, this study sought to create a ‘plug-and-play’ system for production of large amounts of GE. The production process was designed with commercial and industrial foresight, seeking to keep the process as straightforward, safe, and cost effective as possible, while limiting any proprietary restrictions. The use of a constitutive, patent-free, P_GAP_-*Pichia pastoris* (glyceraldehyde-3-phosphate dehydrogenase promoter) expression system, to produce *Hypocrea jecorina* GE at bioreactor scale was investigated. Both the enzyme production itself as well as downstream processing of the enzyme into a stable product were considered (Additional file 1: Fig. S1). The feasibility of this approach was further tested for another emerging enzyme of limited commercial availability, *Neurospora crassa* cellobiose dehydrogenase (CDH). CDH finds application in multiple areas including bioremediation, textile, biomedicine, biosensors and biofuels [15]. CDH could improve the saccharification of pre-treated lignocellulose through reduction of end-product inhibition and catalytic activation of lytic polysaccharide monooxygenase [16]. This type of studies is necessary to facilitate research of the functionality and application of GE and other emerging enzymes such as CDH.

## Main text

### Methods

#### Construction of the production strains

The *P. pastoris* type strain DSMZ 70382 (CBS704) (DSMZ German Collection of Microorganisms and Cell Cultures) was selected as the expression host. The commercial pJexpress 905 (pJ905) vector (ATUM, USA) containing the P_GAP_ and alcohol oxidase I (AOX1) terminator, was used to generate constructs for GE (AAP57749.1) and CDH (XM_951498.2) expression and secretion. Details of cloning, transformation, confirmation and screening procedures are described in [17]. The transformant showing the best production during screening in shake flasks for each enzyme was selected for production at bioreactor scale.

#### Bioreactor cultivations

The inoculum preparation and fermentation procedure were conducted as described in Invitrogen guidelines [18, 19]. Fermentations were conducted in 14 L New Brunswick BioFlo 110 bioreactors, using BioCommand^®^ software (Version 3.30 Plus, New Brunswick Scientific Co. Inc.). The fermentation conditions were identical for all fermentations: 30 °C, pH 5 (combination glass pH probe Mettler Toledo), dissolved oxygen (DO) maintained at 30% (polarographic DOT probe, Mettler Toledo), and an aeration rate of 1 vvm. The fed-batch stage was initiated after depletion of glycerol, where 4 L of 50% (w v^−1^) glycerol feed, supplemented with PTM_1_ trace salts solution (1.2% v v^−1^), was fed at a constant rate of 72.6 mL h^−1^. The fed-batch stage was concluded after approximately 48 h, when the feed was depleted.

Samples taken (10 mL) during fermentation were analysed for biomass (dry cell weight, g_DCW_ L^−1^) enzyme activity (U L^−1^) as described in [17, 20, 21], and glycerol concentration [22] (during fed-batch stage). The final samples were used to determine total protein concentrations [bicinchoninic acid (BCA™) microassay (Sigma-Aldrich, USA)] and target protein concentrations [densitometry of tris-tricine sodium dodecyl sulphate polyacrylamide gel electrophoresis (SDS-PAGE) [23] image using ImageJ^®^ software]. Maximum growth rate (μ_max_) was graphically calculated during the exponential growth phase of the batch phase. All trends were fitted to a linear regression with R^2^ values of above 0.98.

#### Concentration of crude enzyme extracts

The culture was harvested immediately after the fed-batch phase to minimize protease degradation of the enzyme product. The total culture was centrifuged (8000 rpm; 4 °C; 10 min) and subjected to a two-step filtration process. The Pellicon 2.0 tangential flow filtration apparatus (Merck, South Africa) was used as per the manual instructions. The feed and retentate pressure were maintained at a maximum of 10 and 2 bar, respectively. The transmembrane pressure (TMP) was 2.5 bar. The supernatant was first filtered through a 0.22 μm filter cassette (Durapore^®^ PVDF, Merck Millipore), then concentrated by ultrafiltration using a 5 kDa filter cassette (Biomax™ 5, Merck Millipore). Once the volume of the retentate/feeding reservoir was decreased tenfold, the process was complete. Samples of each permeate and retentate were analysed for volumetric activity. Volumes of the feed, permeate and retentate of each filtration process were measured.

### Results and discussion

#### Glycerol fed-batch fermentation kinetics, yields and productivity for GE and CDH

**Fig. 1:**
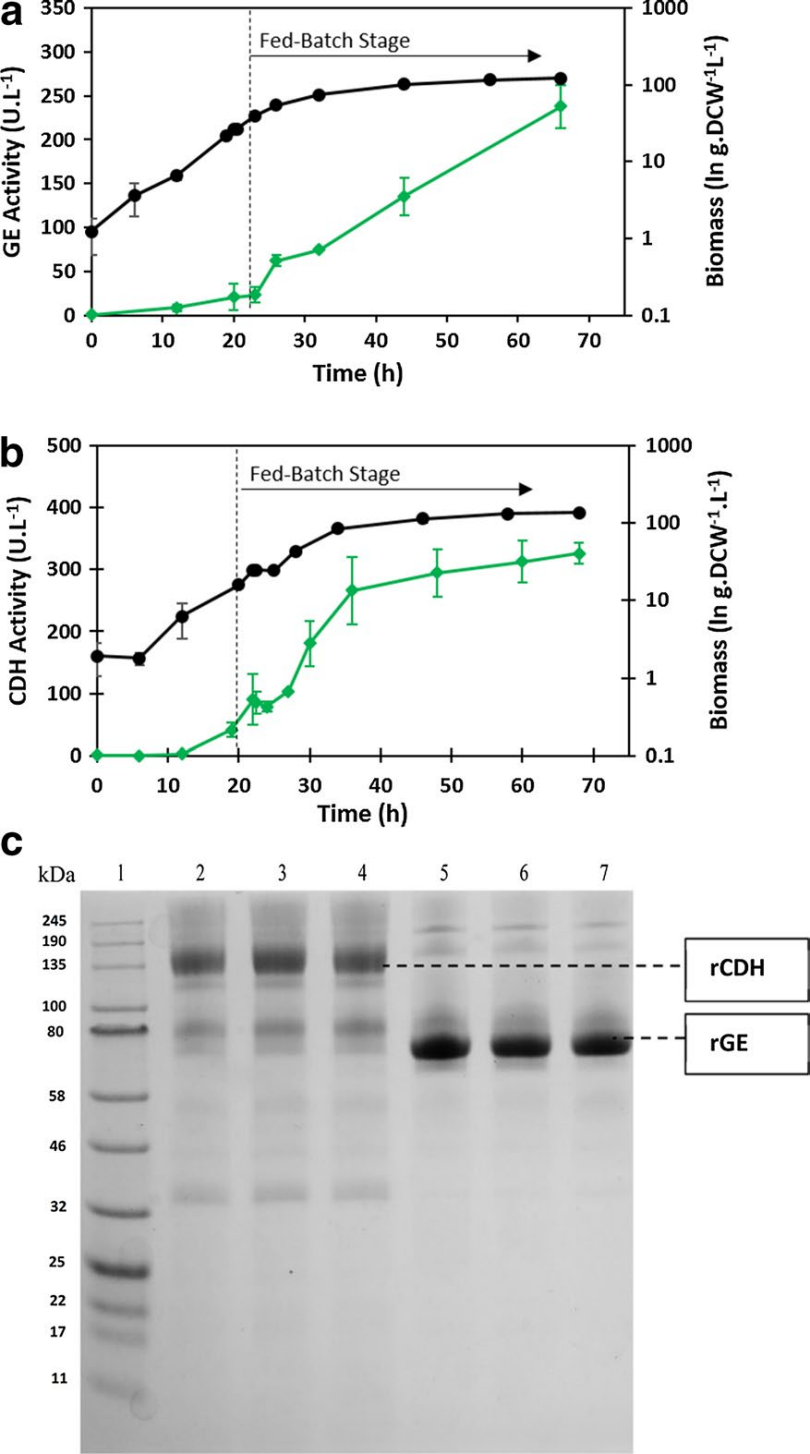
Volumetric activity (green diamond) and biomass yields (dry cell weight per litre) (black circle) during bioreactor cultivations of each target protein. Arrows indicate the initiation of the glycerol fed-batch phase (20–22 h), where a 50% (w.v^−1^) glycerol feed supplemented with trace salts was added at a rate of 72.6 mL/h. **a** Glucuronoyl esterase (GE) **b** Cellobiose Dehydrogenase (CDH). Error bars indicate standard deviation from mean of three fermentation replicates. **c** SDS-PAGE analysis of final fermentation samples (each lane representing one replicate of a bioreactor fermentation) of 20 μL supernatant containing the target proteins. Lane 1: Protein molecular weight marker. Lane 2–4: CDH-containing supernatant (rCDH, 127 KDa). Lanes 5–7: Glucuronoyl esterase-containing supernatant (rGE, 78 KDa)

The growth and enzyme production characteristics of the created recombinant strains were evaluated using bioreactor cultivations. GE side-activity of three commercial enzyme cocktails, namely Celluclast^®^ 1.5 L (Novozymes, 27.47 U L^−1^), Depol™ 740 L A (Biocatalysts, 10.70 U L^−1^) and Depol ™ 740 L B (Biocatalysts, 7.72 U L^−1^) were tested to serve as a comparative baseline. The bioreactor cultivation for expression of GE returned a final volumetric activity yield of 238.17 U L^−1^, and a final recombinant protein titre of 2778.01 mg L^−1^ (Fig. 1a; Table 1), well in excess of previous attempts involving methanol induction of GE [5, 24]. Notably, the volumetric activity was well above the side activities of the commercial enzyme cocktails tested here (7.72–27.47 U L^−1^). Bioreactor cultivation for CDH production returned a final mean volumetric activity of 329.49 U L^−1^ and a final mean recombinant protein concentration of 1489.30 mg L^−1^ (Fig. 1b; Table 1). Although the protein concentration of secreted CDH reported here (1489.30 mg L^−1^) is an improvement on previous attempts at methanol-induced expression, the volumetric activity and specific activity is lower than previously reported [21, 25–27]. Lowered specific activity has been observed in recombinant CDH production by *P.* *pastoris* due to a sub-stoichiometric occupation of catalytic sites within the flavin adenine dinucleotide (FAD) cofactor, as well as hyper-glycosylation [28]. Further, hyper-glycosylation may have affected the specific activity of both target enzymes, since the reported protein sizes (Fig. 1c; CDH: 127 kDa; GE: 78 kDa) are larger than the expected, in silico protein sizes (ExPASy: CDH: 88.46 kDa; GE: 81.29 kDa) [2, 29].

**Table 1:** Summary of enzymatic yields, kinetics and productivity for bioreactor cultivations of recombinant *P. pastoris* at the end of the glycerol fed-batch stage, and comparisons to previous expression attempts

Parameter	GE (this study)	GE (previous studies)	Reference	CDH (this study)	CDH (previous studies)	Reference
Highest Volumetric Activity (U L^−1^)	238.17	5.7^a^	Topakas et al. [5]	329.49	7955^a^	Stapleton et al. [26]
Highest Recombinant Protein Titre (mg L^−1^)	2778.01	NR	–	1489.30	633^b^	Ma et al. [28]
% recombinant protein vs total protein in supernatant	93.61%		–	50.89%	NR	–
Biomass concentration (g_DCW_ L^−1^)	120.94		–	136.47		–
μ_max_ (h^−1^)	0.15		–	0.16		–
q_p.max_ (mg recombinant protein.[g biomass∙h]^−1^)	1.22		–	0.76		–
q_p.mean_ (mg recombinant protein.[g biomass∙h]^−1^)	0.52		–	0.43		–

*NR* Not reported

^a^At shake flask scale, under methanol-induced P_AOX1_ regulation

^b^At 7 L bioreactor scale, under methanol-induced P_AOX1_ regulation

The general trends observed for the fermentations are as follows (Fig. 1a, b). During the batch phase, an initial lag phase of less than 6 h was observed, corresponding to minimal volumetric activity, followed by an exponential growth phase, where linear increases in volumetric activity were observed until the end of the batch phase (between 20 and 22 h). During the glycerol fed-batch stage, there was a brief exponential growth phase of approximately 6 h, followed by an extended growth phase. During this continuous growth phase, the agitation of the bioreactor remained at the maximum (1000 rpm), and oxygen sparging was required throughout to maintain sufficient availability of DO (30%) in the culture.

The μ_max_ was determined during the batch phase under identical growth conditions (Table 1). GE- and CDH-expressing *P. pastoris* strains had similar μ_max_ values of 0.15 h^−1^ and 0.16 h^−1^ respectively, within expected values reported for recombinant *P. pastoris* strains using glycerol (0.15–0.20 h^−1^) [30, 31]. Similarly, the biomass yields obtained (120.94, 136.47 g_DCW_ L^−1^; Table 1) were comparable to previous bioreactor cultivations of recombinant *P. pastoris* [18, 32].

A decrease in specific growth rate, modelled as a power regression (GE: 20979x^−3.63^; R^2^ = 0.9871; CDH: 885.76x^−2.852^; R^2^ = 0.9882), was observed during the fed-batch process (Additional file 2: Fig. S2), during which the levels of glycerol remained below detectable HPLC levels. This is attributed to the fact that the amount of biomass in the fermentation vessel increased with time (Additional file 2: Fig. S2), while the amount of glycerol fed into the bioreactor remained constant, effectively decreasing the ratio of available carbon source per gram of biomass. This growth rate decline has been suggested to be the concluding stage of an ideal growth rate time course for a number of secreted protein fermentations in *P. pastoris* [30, 33, 34].

Specific productivity describes the efficiency of the process by quantifying the amount of recombinant enzyme secreted per gram of biomass per hour. Maximum values of specific productivity (q_p.max_) of protein secretion were reached during the batch phase (1.22, 0.76 mg recombinant protein.biomass^−1^ h^−1^ for GE and CDH, respectively; Table 1). During the fed-batch stage, specific productivity remained constant at a lowered value close to the mean specific productivity value (q_p.mean_) (0.52 and 0.43 mg recombinant protein.biomass^−1^ h^−1^ for GE and CDH, respectively), resulting in GE and CDH accumulation throughout the fed-batch stage (Fig. 1a, b). This has been observed in previous constant fed-batch cultivations of recombinant P_GAP_-*P. pastoris* [33, 35]. The productivity could be increased by manipulating the growth rate such that an extended growth phase at μ_max_ is maintained before the end-phase decline in growth rate [31, 33–35]. This is achieved by an increasing (linear or exponential) glycerol feeding rate during the fed-batch stage. The optimal duration of the phase at which μ_max_ is maintained differs between recombinant strains and should be optimised on a case-by-case basis.

#### Concentration of crude enzyme extracts by tangential flow ultrafiltration (TFU)

The final secreted protein concentrations differed between enzymes, probably due to protein-specific factors associated with the secretion process. The heterologous proteins were secreted at moderate (CDH: 50.89%) to high (GE: 93.61%) percentages of the total extracellular protein, as determined by densitometry analyses (Fig. 1c). CDH and GE were, therefore, the major proteins present in the fermentation broth. While native, extracellular *P. pastoris* proteins were present in the fermentation broth (Fig. 1c), these were devoid of any contaminating enzymatic activity (as determined with the use of a negative control strain), which allows for simple and economical downstream processing, contributing to the affordability and accessibility of the process.

The harvested supernatant was concentrated using TFU (Table 2). The microfiltration process returned satisfactory volumetric activity yields (GE: 86.84%, CDH: 86.54%; Table 2), and the loss in activity units can be explained by the inherent loss of sample volume in the filtration apparatus itself. The total protein concentration in the enzyme extracts was increased 3.69-, 2.84-fold for GE and CDH, respectively. In terms of volumetric activity, GE and CDH were concentrated 4.95-, and 5.20-fold, respectively. The ultrafiltration process returned volumetric activity yields that were acceptable (49.57 and 50.00%), but lower than expected [26, 36–38] despite acceptable integrity of membrane (permeate with minimal volumetric activity 0.00–2.95 U_Total_ L^−1^). It is therefore hypothesised that the recombinant protein is being retained or degraded in the filtration apparatus itself, as proteins can adsorb to membranes, often causing permanent fouling [39] and the formation of a protein monolayer on the surface of the membrane [40]. Despite these shortcomings, the final concentrated enzyme products could be used directly on lignocellulose according to enzyme dosages established in previous studies [2, 41].

**Table 2:** Summary of volumetric activity and protein concentration values at successive steps of tangential flow filtration concentration of cell-free extracts obtained from bioreactor fermentations

	Step	Volumetric activity (U L^−1^)	Total activity (U)	Yield (%, volumetric activity)	Total protein concentration (g L^−1^)
GE	CFE	203.92	407.84	100.00	2.93
	MFP	196.07	352.93	86.54	–
	UFR	1019.61	203.92	50.00	8.31
	UFP	2.11	2.95	–	–
CDH	CFE	40.77	81.54	100.00	2.97
	MFP	39.34	70.81	86.84	–
	UFR	202.08	40.42	49.57	10.16
	UFP	0.03	0.04	–	–

*CFE* cell-free extract, *MFP* microfiltration permeate, *UFR* ultrafiltration retentate or concentrated enzyme, *UFP* ultrafiltration permeate

## Limitations

This is the first report of GE secretion yields under constitutive expression, and the second report of bioreactor heterologous production [9]. This work demonstrates the feasibility of a constitutive, methanol-free *P. pastoris* expression system for production of emerging lignocellulose-degrading enzymes such as GE. The bioprocess reported here returned high protein yields of active GE and shows promise for different enzymes as well, including CDH (Additional file 1: Fig. S1). However, the constant specific productivity and the lack of an extended μ_max_ phase during the fed-batch cultivation shows potential for improvement of secretion levels through manipulating the growth rate. TFU concentrated the enzyme extracts to suitable dosages for lignocellulose application studies but showed inefficient volumetric activity yields. This is significant due to the economic implications at manufacturing scale, therefore optimization of the ultrafiltration process is recommended. Future studies should also confirm the application of the concentrated recombinant enzymes on industrially relevant substrates.

## Supplementary information

**Additional file 1: Figure S1.** Illustration showing the strategy applied for the production of glucuronoyl esterase (GE) and cellobiose dehydrogenase (CDH) enzymes and the summary of main results.

**Additional file 2: Figure S2.** Exponential decline in specific growth rate during glycerol fed-batch stage of the bioreactor cultivation, with a constant glycerol feed of 72.6 mL/h. of (A): Glucuronoyl esterase; (B): Cellobiose dehydrogenase.

## Data Availability

Tabulated data of graphs reported in this study are available in Ref. [17] (Addendum D): https://pdfs.semanticscholar.org/4a02/774e4d4f01751b0140a315b617bb36d233ff.pdf.

## Abbreviations

AOX1: alcohol oxidase 1
CDH: cellobiose dehydrogenase
CFE: cell-free extract
DO: dissolved oxygen
DCW: dry cell weight
GE: glucuronoyl esterase
MFP: microfiltration permeate
P_GAP_: glyceraldehyde-3-phosphate dehydrogenase promoter
qp._max_: maximum specific productivity, mg recombinant protein.biomass^−1^ h^−1^
qp._mean_: mean specific productivity, mg recombinant protein.biomass^−1^ h^−1^
SDS-PAGE: sodium dodecyl sulphate polyacrylamide gel electrophoresis
TFU: tangential flow ultrafiltration
TMP: transmembrane pressure
UFP: ultrafiltration permeate
UFR: ultrafiltration retentate or concentrated enzyme
U L^−1^: units of enzymatic activity per litre
vvm: volume per volume per minute
μ: specific growth rate
μ_max_: maximum specific growth rate
