# Supplementary figures and images for: Scalable methanol-free production of recombinant glucuronoyl esterase in Pichia pastoris
Source: BMC Res Notes. 2019 Sep 18;12:596. doi: 10.1186/s13104-019-4638-9 (PMC6751620; doi:10.1186/s13104-019-4638-9)

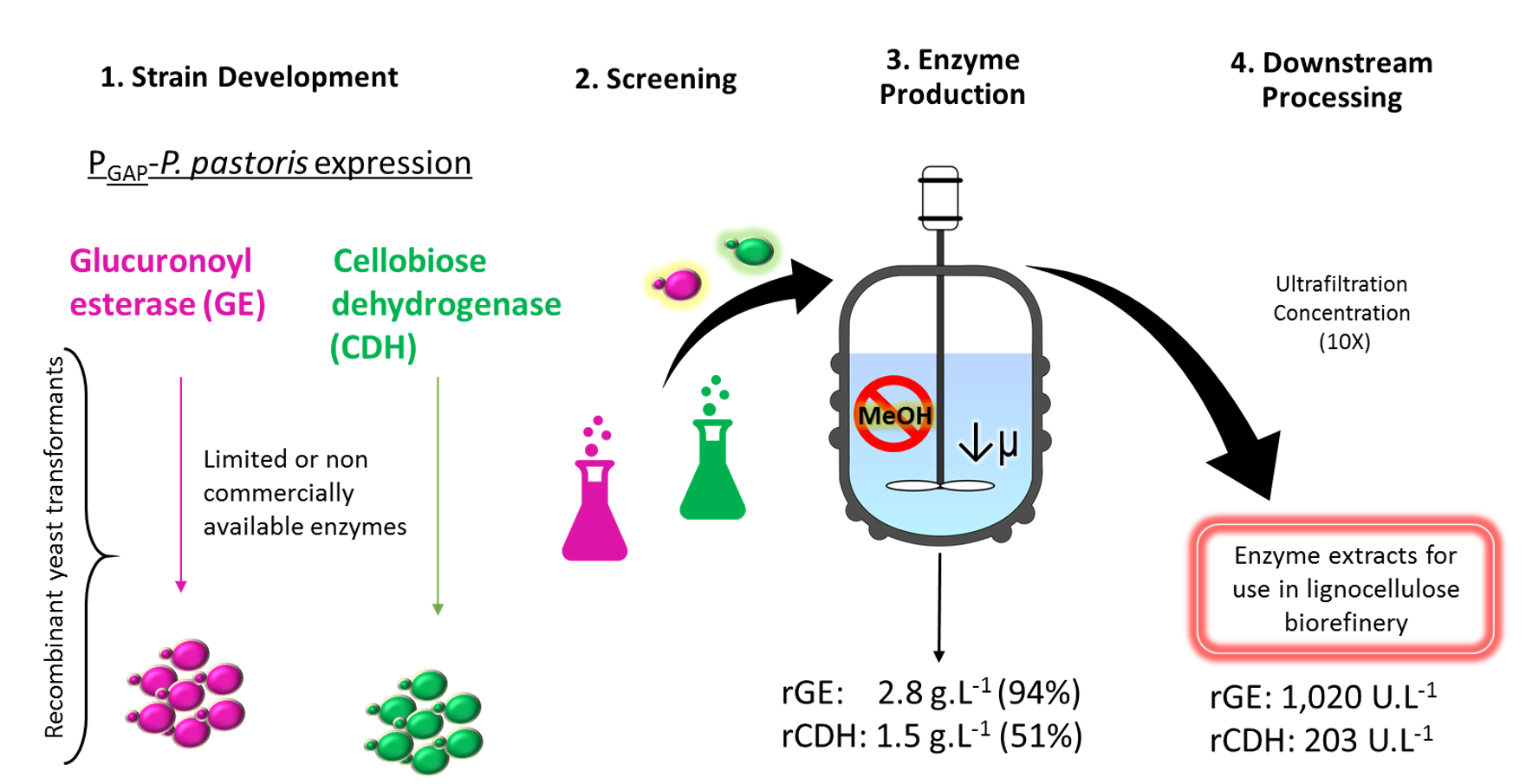

Supplement: Supplementary file 1 — Additional file 1: Figure S1. Illustration showing the strategy applied for the production of glucuronoyl esterase (GE) and cellobiose dehydrogenase (CDH) enzymes and the summary of main results. [file 13104_2019_4638_MOESM1_ESM.tiff]

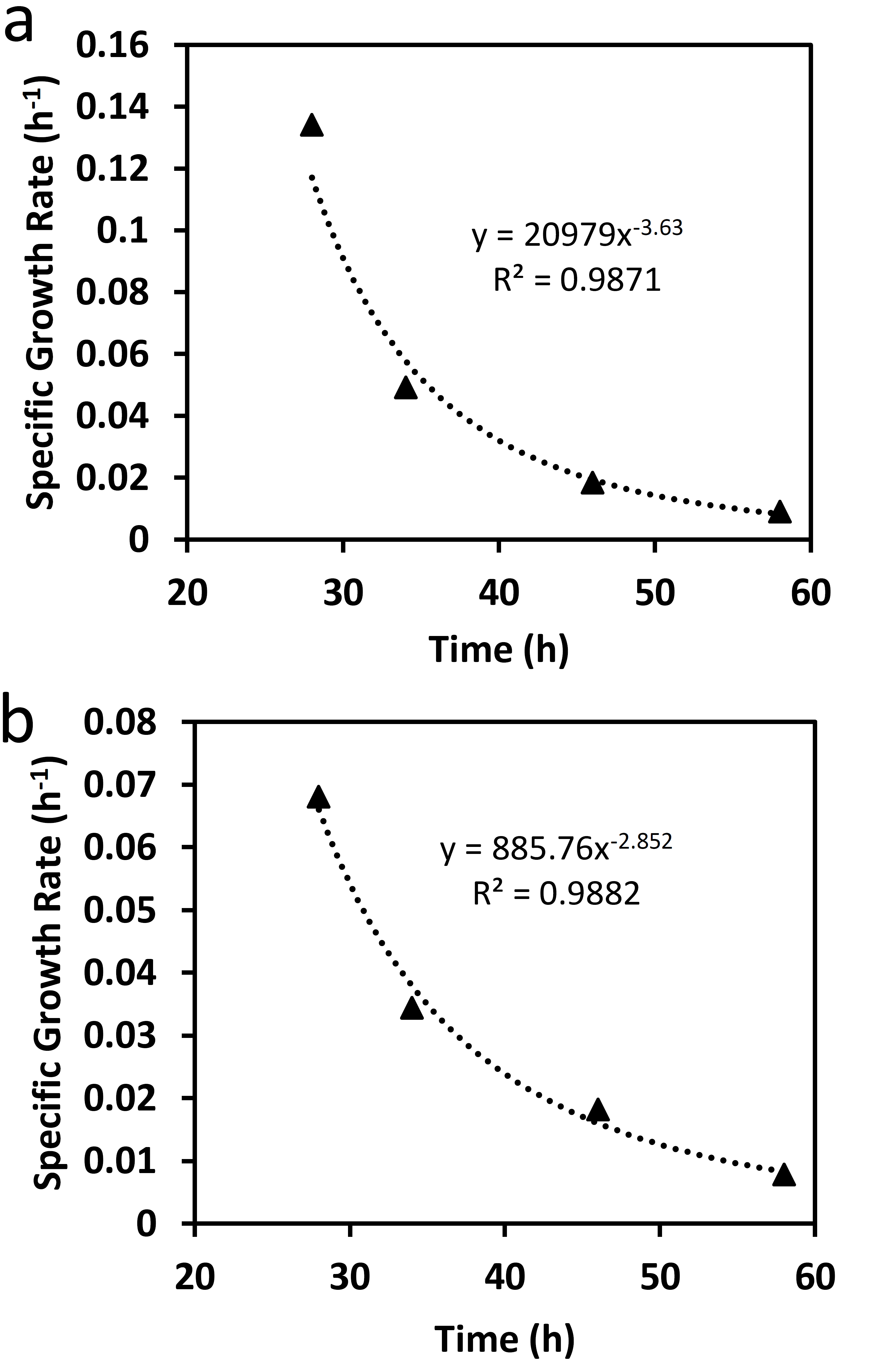

Supplement: Supplementary file 2 — Additional file 2: Figure S2. Exponential decline in specific growth rate during glycerol fed-batch stage of the bioreactor cultivation, with a constant glycerol feed of 72.6 mL/h. of (A): Glucuronoyl esterase; (B): Cellobiose dehydrogenase. [file 13104_2019_4638_MOESM2_ESM.tiff]
